# Perinatal palliative care: focus on comfort

**DOI:** 10.3389/fped.2023.1258285

**Published:** 2023-09-26

**Authors:** F. T. McCarthy, A. Kenis, E. Parravicini

**Affiliations:** Department of Pediatrics, Division of Neonatology, Columbia University Irving Medical Center, New York, NY, United States

**Keywords:** comfort care, perinatal palliative care, neonatal palliative care, end-of-life, life-limiting condition

## Abstract

Providing comfort while a patient is living with a life-limiting condition or at end of life is the hallmark of palliative care regardless of the patient's age. In perinatal palliative care, the patient is unable to speak for themselves. In this manuscript we will present guidelines garnered from the 15-year experience of the Neonatal Comfort Care Program at Columbia University Irving Medical Center, and how they provide care for families along the perinatal journey. We will describe essential tools and strategies necessary to consider in assessing and providing comfort to infants facing a life-limiting diagnosis *in utero*, born at the cusp of viability or critically ill where the burden of care may outweigh the benefit.

## Introduction

Almost 15,000 neonatal deaths are reported every year in the United States, with congenital anomalies being the leading cause of demise (1). Because of advances in prenatal diagnosis techniques, most infants affected by life-limiting conditions, are identified before birth. Moreover, a significant number of newborns admitted to the neonatal intensive care unit in critical condition face potentially adverse prognoses.

Perinatal Palliative Care (PPC) programs are interdisciplinary coordinated services offered to women who elect to continue the pregnancy with a life-limiting or life-threatening fetal diagnosis from the time of diagnosis through the neonatal period and beyond (2). PPC offers a plan of care for improving the infant's quality of life when the patient's prolongation of life is no longer the goal of care, or the complexity of the medical condition is associated with uncertain prognosis (3).

The number of PPC services has been increasing nationally and internationally and recommendations for management of infants with life-limiting or life-threatening conditions have been published (4–9). Proposed guidelines highlight the complexity of the medical needs of these infants and “comfort” is primarily assessed with the help of pain scores. However, a state of comfort encompasses much more than a state of “lack of pain”, and it is better defined as “the satisfaction of the patient's basic physiologic and psychosocial needs”.

The Neonatal Comfort Care Program (NCCP) is a service of PPC at Columbia University Irving Medical Center (CUIMC) that started in 2008 out of a desire to provide a safe, comfortable, and loving environment for infants diagnosed with life-limiting conditions and their families (10). Specifically, the program focuses on assessment and achievement of each infant's state of comfort (11).

The purpose of this article is to share guidelines developed during the 15-year experience of the NCCP at CUIMC. Thus, we provide practical information for professionals interested in the implementation of original guidelines aimed at ensuring a state of comfort to infants with life-limiting or life-threatening conditions by using our model.

## NCCP’s guidelines focused on comfort

The NCCP at CUIMC is a multidisciplinary team led by a neonatologist who serves as medical director. The core team is dedicated to the program and includes a nurse coordinator, nurse midwife, social worker, and manager. The team works closely with maternal-fetal-medicine and neonatology teams and other professionals (a psychologist, speech pathologist, lactation consultant, Child Life specialist and chaplain) who serve as consultants. The program's origin, growth and organization have been previously described (11).

The team established and implemented original guidelines based on the 8 palliative care domains proposed by the Clinical Practice Guidelines for Quality Palliative Care (12). The essential strategies aimed to fulfill the infant's basic physiologic and psychosocial needs include bonding, maintenance of body temperature, relief of hunger/thirst and alleviation of discomfort/pain (13). Tables 1, 2 offer detailed clinical suggestions for different clinical scenarios.

**Table 1:** Tasks to provide comfort to newborns with life-limiting conditions in the perinatal period.

Before delivery
Review birth plan (prepared with family during pregnancy) including:
• Medical plan: need to obtain genetic or other testing to confirm diagnosis
• Supportive Plan: involve multidisciplinary services (^a^)
Collaborate with nursing leadership to obtain private room and coordinate visitation
Gather supplies: camera, clothing, memory box, items for medical dressings, if needed
After delivery
Dry, suction, quickly assess newborn's clinical features and status to confirm prenatal findings: it can be done at the radiant warmer or while skin-to-skin with mother
Place hat, diaper and clean blanket for warmth
If a special medical dressing is needed bring newborn to radiant warmer to apply dressing
Encourage skin-to-skin (cheek-to-cheek in case of cesarian delivery) or holding
The newborn should stay with the family unless they request otherwise to facilitate bonding
Coordinate interventions by multidisciplinary services (^a^) and be aware of cultural and religious expectations including desired rites or rituals
Offer interventions according to the newborn's clinical status:
• Encourage skin-to-skin or holding
• Consider methods of feeding: colostrum care, breastfeeding, bottle, syringe
• Assess for discomfort/pain and treat accordingly including non-pharmacological strategies (gentle suctioning upper airways, positioning) and/or pharmacological treatment (acetaminophen PO/ PR; morphine sulfate PO; fentanyl IN; lorazepam PO; midazolam IN)
• The newborn should be transferred with the mother to post-partum in accordance with the family's wishes
• If the newborn dies in the delivery room, bonding and memory making are still encouraged
• If desired, the family can bring the newborn's body with them to post-partum
• Discuss with the family option for autopsy or other post-mortem testing to confirm diagnosis
Post-partum course
Provide a private room and facilitate bonding, family involvement and memory making
Assess pain and discomfort: use approved pain score, note any change in clinical status and respiratory effort, note parental observations
Provide privacy and minimal interruptions
Medical orders:
• Do Not Resuscitate/Do Not Intubate (DNR/DNI)
• Vital Signs: heart rate, respiratory rate, temperature and pain score every 12 h and as needed
• Avoid blood or other testing, unless needed for confirmation of diagnosis
• Discuss whether to obtain Newborn Screening Test and administration of Vitamin K and eye prophylaxis
• Nutrition: feed breastmilk and/or formula ad lib; discuss with the family a plan for nutrition focused on comfort by avoiding hunger/thirst. Consult specialists as needed such as lactation consultant for feeding and breast care and speech language pathologist if the newborn is unable to breast or bottle feed
• Discuss discharge options (home hospice or transition to rehabilitation for hospice care)

PO: per os; PR: per rectum; IN: intranasal.

^a^ Social worker, child-life specialist, chaplain, psychologist.

**Table 2:** Tasks to provide comfort to NICU infants with life-threatening or terminal conditions.

Critically ill infants receiving full intensive care
Integrate clinical status and strategies for comfort interventions into discussions with primary medical and nursing care teams
Include parents in assessment and conversations regarding plans for comfort interventions
Involve multidisciplinary services (^a^) and be aware of cultural and religious expectations including desired rites or rituals
Provide privacy, if possible, and facilitate bonding:
• Encourage parents’ participation in daily cares (diaper change, feeding, bathing, dressing)
• Provide opportunities for holding and therapeutic touch
• Teach alternative methods for comfort and bonding: colostrum care, non-nutritive strategies, massage, reading, music, memory making
• Include siblings and extended family in accordance with the family's wishes
Infants at the end-of-life stage after redirection of goals of care
Create plan for removal of life-sustaining support and pain management with parents, medical and nursing teams, assuring that comfort can be achieved
Involve multidisciplinary services (^a^) for important rites/rituals, memory making, emotional support, funeral planning
Do not resuscitate/do not intubate (DNR/DNI) and pain medication orders
Provide privacy and facilitate bonding:
• Turn off monitors
• Pull tubes, wires, arterial or venous lines, but maintain IV access for pain management
• Encourage skin-to-skin and holding
• Consider alternative methods for comfort and bonding: colostrum care, non-nutritive strategies, massage, reading, music
• Include siblings and extended family in accordance with the family's wishes
Pain assessment and management:
• Assess respiratory distress (air hunger, agitation, increased work of breathing, gasping) and treat with nonpharmacological strategies (gentle suctioning upper airways, positioning) or pharmacological treatment (morphine sulfate PO/IV; fentanyl IN; lorazepam PO/IV; midazolam IN)
• Assess pain by validated clinical scores and treat with nonpharmacological strategies (positioning, skin-to-skin, sucking) or pharmacological treatment (acetaminophen PO/PR/IV; morphine sulfate PO/IV; fentanyl IN; lorazepam PO/IV; midazolam IN)
Assist family with care of the body after death: holding, bathing, dressing
Discuss with the family option for autopsy or other post-mortem testing to confirm diagnosis

PO: per os; IV: intravenous; IN: intranasal; PR: per rectum.

^a^ Social worker, child-life specialist, chaplain, psychologist.

### Bonding

Human beings are social creatures and the need for connectedness and relationship is inherent. According to developmental psychiatrists, humans are biologically “prewired” for connection even before birth (14). Within PPC, the process of attachment holds great importance although the physical connection may be brief. Fostering this connection between infant and parent as well as bonding with siblings and extended family is implemented in the delivery room, postpartum unit, and Neonatal Intensive Care Unit (NICU).

In the delivery room an infant born with a life-limiting condition is put on the mother's chest as soon as possible. Alternatively, the infant can be brought to a warmer first, dried with warm towels, and quickly assessed. The cord can be cut by the partner if desired, a hat and a diaper are placed and then the infant is returned to the mother's chest.

After birth by cesarean section, the newborn is brought to the mother and placed cheek to cheek so that she can see, hear, smell, and touch her infant. The partner or support person can hold the infant on the mother's chest or next to mom so that she can see the infant easily. The mother is recovered in a private labor room to allow privacy.

Newborns are bulb suctioned, if needed, while on the warmer or while being held skin-to-skin. For infants with severe anomalies such as anencephaly or gastroschisis, dressings are applied before returning the infant to mom. This facilitates holding the newborn comfortably. Multiple pictures of the infant alone and of the whole family are taken.

Considerations are made about the infant's physical differences when taking pictures. Usually, one picture is taken with a newborn undressed to show anomalies. For some parents, the fantasy about what their infant's anomaly might look like is worse than what it does look like. In other situations, it serves to show exactly why the infant will have a short life. This photo is printed, placed in an envelope, and kept separate from other printed photos. This gives families a choice of whether to look at the photo in the future or discard it.

Coordination with nursing leadership is done to allow for sibling and family visitation. Cultural and religious considerations are also very important. Families are asked about rites, rituals, and customs so that staff can optimize the plan of care respecting family preferences (15). Collaboration with a multidisciplinary team is key for family satisfaction.

This is a very important time for the family. It may be the only time they spend with their infant while he/she is alive. Parents are encouraged to bond with their infant by skin-to-skin holding, talking, singing, praying or anything that is important to the family. Staff prepares the family for physical changes that may be seen as the infant is dying. Changes in the infant's breathing, color and temperature are presented to the family in a calm concrete manner, assuring them that the infant will be assessed for pain and medicated if needed.

When mom is discharged to postpartum her infant remains with her. Families are given private rooms that enable them to spend time with their infant, performing parenting tasks such as holding, bathing, dressing, and taking pictures. Feeding, when possible, is encouraged as part of the bonding process (16).

In the case of a stillbirth or intrauterine fetal death, time for bonding is also important. Under these circumstances, the infant's body may have begun to deteriorate, and parents are counseled about this possibility, but without judgement as to whether the infant's body should be seen. Families may ask for staff to view the infant first or take a picture before actually seeing the infant. Allowing the family time to process their feelings affords them the opportunity to make decisions. If they initially decline seeing their infant, they may need more time. The actions of the staff toward the body can help families feel empowered to view and possibly hold their infant. Some families want to see, hold, and dress their infant no matter what their condition is. Staff remains flexible to meet the family's needs.

Families are not rushed and can keep the infant with them throughout the mother's hospital stay if desired. This is the only time they will have to parent their infant and they are encouraged to hold, bathe, dress and photograph their infant as they would have done with any other child. Cooling systems are available commercially but are not required for a body to remain in the mother's room. The amount of time a family will keep the body varies greatly. Staff education has given them the theory and strategies to help support families in meeting their desires.

While intensive care offers many infants’ curative treatments it does, by nature, interrupt parent-child bonding, thus bonding in the NICU can be challenging. Issues with lack of privacy, and the sound of monitors and alarms makes it difficult to create a calm and quiet environment. Some degree of bonding is indeed always possible. Parents are given the opportunity to help with their infants’ medical needs such as taking the temperature, feeding, changing the diaper, and helping with medical dressing. Infants in the NICU can receive intensive care and palliative care concurrently as palliative care is thought of as an additional layer of support. When, during the NICU course, there is redirection of goals of care, private space is made available and signage alerts others to be sensitive to the situation that is unfolding or that an infant has died. Redirection of care entails a plan made by the medical team with the family. The details include a timeline, religious rites or rituals, sibling and extended family visitation, memory-making, medication management of symptoms and help from consulting services (i.e., consulting surgery for removal of the ECMO circuit). Skin-to-skin holding greatly facilitates the bonding experience. NICU nurses have shared that utilizing skin-to-skin holding of a dying infant afforded an opportunity for parents to experience their infant's closeness and touch, providing comfort without the tubes and wires (17).

Bedside staff help in any way the family needs. Infants can be bathed and dressed before or after death. Some families may be hesitant to bathe the infant after he/she has died but staff help to normalize this activity by explaining the process, gathering supplies, and assisting. Reverence in caring for the body communicates respect and recognition of the importance of the infant to the family. Moreover, it is an opportunity to see their child's body without lines and their child's face free from tubes (18).

### Maintenance of body temperature

Another essential element to provide comfort to an infant is keeping them warm and dry. Again, one of the easiest ways to accomplish this is through skin-to-skin holding. Often utilized in the NICU, skin-to-skin holding has several physiological benefits including providing thermal regulation (19) and can be done by mothers or fathers. Research shows that skin-to-skin holding can maintain a stable temperature in full term and very low birth weight premature infants (19, 20), thus is utilized even with pre/periviable newborns. While in the postpartum unit, an incubator or a warmer can be used for infants having difficulty maintaining their temperature especially if parents need to have a safe place for their child while they rest.

### Relief of hunger/thirst

In recognition of the fact that feeding is a basic need and a pleasure for the infant and reinforces bonding, infants are offered breast milk or formula. Feeding for the comfort of the infant and the parent fulfills an instinctual desire to provide nourishment because this is associated with life (16). The American Academy of Pediatrics (AAP) recommends feeding any child that is hungry or thirsty and who is capable of oral intake, when there are no medical contraindications, to be fed by mouth (21). The AAP also shares that the provision of medically provided nutrition and hydration is not ethically or legally required in many life limiting medical conditions, and parents have choices (21). However, in our 15-year experience, feedback from parents indicate that feeding constitutes a positive experience for both infant and parent (22, 23). Within the framework of palliative care, feeding should be focused on comfort, but not necessarily on growth and development. At this aim the medical team discusses with the parents a personalized plan for nutrition according to each infant's diagnosis, prognosis, and family preference. Even when life is short, the experience of feeding her child by breast or bottle can be extremely important to the mother. Lactation consultants offer guidance and techniques to help mothers recognize feeding cues, help newborns latch, teach mothers to hand express milk, and perform breast care.

When nutrition is an option and it is safe, infants who have muscle weakness, small mouths, or physical anomalies such as a cleft lip or palate can be evaluated by a speech pathologist. Special devices are used based on their assessment of the infant's feeding readiness to allow for safe and pleasurable feeding. If a newborn is unable to manage oral feeds, a nasogastric or orogastric tube or, if needed, a gastrostomy tube is placed. Some families learn to give feedings via a syringe or dropper. Bedside nurses provide support and education as the parents learn to use the devices for feeds. The focus of care in these instances is a balance between comfort and safety.

For infants in state of impending death drops of colostrum or breastmilk can be swabbed on their lips and mouth (colostrum care). Offering a pacifier, emptied breast or gloved finger can be used as non-nutritive options.

In the NICU, when there is a redirection of care, withholding intravenous nutrition is acceptable. In these situations, intravenous access is maintained for medicine administration to alleviate pain or discomfort.

For mothers whose infants have died, lactation consultants educate them about how to suppress their milk supply. However, some mothers may choose to become milk donors and information is given to them about the process and contact information for a nearby milk bank.

### Alleviation of discomfort/pain

The greatest concern parents have in the face of a life-limiting condition in their infant is, will my child suffer? While most congenital life-limiting conditions with expected early demise such as anencephaly or renal agenesis are not painful, symptoms of the dying process can be distressing. Parents can be assured that all the components of their infant's comfort will be addressed. Since comfort is not merely the lack of pain, all the previously mentioned strategies are utilized first. Air hunger, gasping, increased work of breathing or agitation, can sometimes be relieved by gentle suctioning or repositioning. When non-pharmacological strategies are unsuccessful, pharmacological treatment is necessary. Opioids and benzodiazepines can be used orally or intranasally with no need of placing intravenous access. The assessment of pain or discomfort is evaluated by medical and nursing staff using approved pain scales (24) but reports by the family of discomfort are addressed immediately as well.

Many infants have physical anomalies possibly producing discomfort or pain. Depending on the anomaly, special dressings are needed for comfort. For instance, infants with limb-body-wall syndrome or with other conditions associated with exteriorization of internal organs will have a surgical dressing. Warm saline soaked gauze, covered with dry gauze and plain petroleum jelly covered with an elastic bandage will contain and protect the organs. Infants with acrania, exencephaly, or anencephaly will need a similar dressing that is covered by a hat that ties under the chin to keep the dressing intact. Despite having anomalies, staff will also point out aspects that are ‘perfect’ or ‘beautiful’ about the infant, recognizing the normal physical traits as well.

For families taking their infant home with hospice, medical and nursing staff will educate them about changes that can occur as the infant nears end-of-life, about signs and symptoms of distress, and when to call hospice with concerns. The around-the-clock coverage of hospice is reinforced giving parents a sense of continued support. A Medical Order for Life-Sustaining Treatment (MOLST) form is filled out to document family's preference concerning life-sustaining treatments for the infant.

Lastly, pain management is an essential component of end-of-life care of infants with a life-threatening condition failing treatment in the NICU. Recommendations for assessment and management of end-of-life symptoms in detail would require an entire manuscript and is beyond the scope of this article. Therefore, we invite readers to delve deeper into this subject by reading comprehensive reviews (25–27).

## Discussion

The comfort of the infant is at the center of the care of the NCCP at CUIMC.

The word *comfort* derives from the Latin roots *cum* and *fortis*, the meaning of which (*cum *= with, together; and *fortis *= strong) suggests a relationship that provides strength. The essential elements of comfort measures are relational in nature. The fulfillment of the patient's basic physiologic and psychosocial needs such as bonding, maintaining body temperature, relieving hunger and thirst, and alleviating distress and pain are achieved with strategies based on relationships between the infant, the family, and the medical team (28–31).

A policy statement of the American Academy of Pediatrics provides scientific evidence related to the achievement of a state of comfort in the neonatal population. The use of non-pharmacological strategies such as holding, swaddling, skin-to-skin care, sucking, colostrum care, breastfeeding, formula, or oral glucose has been found to be effective in reducing stress/discomfort in healthy newborns undergoing painful procedures (32). Moreover, Mangat et al. performed a systematic literature search for non-pharmacological pain-treatments and found that most strategies provided some degree of relief to sick newborns treated with intensive care (33).

Individualized, and creative plans of care need to be adapted to each infant's condition, as the strategies for achievement of comfort vary substantially with different clinical scenarios. The needs of a newborn just delivered with a life-limiting diagnosis may be very different from those of an infant admitted to the NICU for months, failing intensive care and approaching the end-of-life stage, as reported in Tables 1, 2.

However, the goal of achieving comfort is always possible when a personalized plan of care is developed considering the diagnosis, the baby's clinical status and the family's preferences. Parravicini et al. assessed the perception of parents concerning the state of comfort in their infants born with life-limiting conditions and treated by the NCCP with strategies focused on comfort. Forty-two parents of 35 infants responded to an anonymous questionnaire with quantitative and qualitative questions. Data analysis showed that parents strongly perceived the achievement of a state of comfort for their infants as a result of the comfort measures (23).

The family is indeed an integral part of the care and strategies aimed at facilitating comfort of the infants provide benefits to their families who experience psychological distress (34). The NCCP developed a model of early palliative care called BACI (baby, attachment, comfort interventions) and offered it to a group of parents of infants admitted to the cardiac NICU with serious conditions. The method included interventions meaningful to parents and aimed at improving the neonates’ quality of life. Exposure to the BACI intervention significantly reduced parental stress (35).

In conclusion, we have described PPC guidelines as they took shape in the NCCP at CUIMC as fruit of a 15-year experience. The program proposes original guidelines aimed at satisfying infants’ basic physiologic and psychosocial needs, thus ensuring a state of comfort to patients with life-limiting or life-threatening conditions at any clinical stage.

We hope that our checklists may help navigate the difficulties and complexity of the perinatal journey, and therefore become the standard of care for each infant with a short life expectancy or with a serious condition and potential adverse prognosis or at the end-of-life stage.

Further studies are needed to verify whether the guidelines proposed by the NCCP are successfully reproducible in other populations and institutions.

## Data Availability

The raw data supporting the conclusions of this article will be made available by the authors, without undue reservation.

## References

[1] Ely DM Driscoll AK. Infant mortality in the United States, 2020: data from the period linked birth/infant death file. Natl Vital Stat Rep. (2022) 71(5):1–18. 10.15620/cdc:120700 36190428

[2] Perinatal palliative care. Committee opinion no. 786. American college of obstetricians and gynecologists committee on obstetric practice, committee on ethics. Obstet Gynecol. (2019) 134:e84–9. 10.1097/AOG.0000000000003425 31441826

[3] Carter BS. An ethical rationale for perinatal palliative care. Semin Perinatol. (2022) 46(3):151526. 10.1016/j.semperi.2021.151526 34839940

[4] Calhoun BC Hoeldtke NJ Hinson RM Judge KM. Perinatal hospice: should all centers have this service? Neonatal Netw. (1997) 16:101–2. 9325884

[5] Ziegler TR Kuebelbeck A. Close to home: perinatal palliative care in a community hospital. Adv Neonatal Care. (2020) 20(3):196–203. 10.1097/ANC.0000000000000732 32384326

[6] Cortezzo DE Ellis K Schlegel A. Perinatal palliative care birth planning as advance care planning. Front Pediatr. (2020) 8:556. 10.3389/fped.2020.00556 33014940 PMC7505922

[7] Czynski AJ Souza M Lechner BE. The mother baby comfort care pathway: the development of a rooming-in-based perinatal palliative care program. Adv Neonatal Care. (2022) 22(2):119–24. 10.1097/ANC.0000000000000838 33783387

[8] Tewani KG Jayagobi PA Chandran S Anand AJ Thia EWH Bhatia A Perinatal palliative care service: developing a comprehensive care package for vulnerable babies with life limiting fetal conditions. J Palliat Care. (2022) 37(4):471–5. 10.1177/08258597211046735 34636715

[9] Locatelli C Corvaglia L Simonazzi G Bisulli M. Paolini L and faldella G “percorso giacomo”: an Italian innovative service of perinatal palliative care. Front Pediatr. (2020) 8:589559. 10.3389/fped.2020.589559 33330283 PMC7710893

[10] *The Neonatal Comfort Care Program*. Available at: www.neonatalcomfortcare.com (Accessed July 8, 2023).

[11] Wool C Parravicini E. The neonatal comfort care program: origin and growth over 10 years. Front Pediatr. (2020) 8:588432. 10.3389/fped.2020.588432 PMC7661470 33194921

[12] National Consensus Project for Quality Palliative Care. Clinical practice guidelines for quality palliative care, 4th Ed. Richmond, VA: National Coalition for Hospice and Palliative Care (2018). Available at: https://www.nationalcoalitionhpc.org/ncp (Accessed July 8, 2023).

[13] Parravicini E Lorenz MJ. Neonatal outcomes of fetuses diagnosed with life-limiting conditions when individualized comfort measures are proposed. J Perinatol. (2014) 34:483–7. 10.1038/jp.2014.40 24651733

[14] Sullivan R Perry R Sloan A Kleinhaus K Burtchen N. Infant bonding and attachment to the caregiver: insights from basic and clinical science. Clin Perinatol. (2011) 38(4):643–55. 10.1016/j.clp.2011.08.011 22107895 PMC3223373

[15] McGuirl J Campbell D. Understanding the role of religious views in the discussion about resuscitation at the threshold of viability. J Perinatol. (2016) 36:694–8. 10.1038/jp.2016.104 27562182

[16] Chichester M Wool C. The meaning of food and multicultural implications for perinatal palliative care. Nurs Womens Health. (2015) 19:224–35. 10.1111/1751-486X.12204 26058905

[17] Kymre IG Bondas T. Skin-to-skin care for dying preterm newborns and their parents—a phenomenological study from the perspective of NICU nurses. Scand J Caring Sci. (2013) 27:669–76. 10.1111/j.1471-6712.2012.01076.x 23016802

[18] Parravicini E McCarthy F. Comfort in perinatal and neonatal palliative care: an innovative plan of care focused on relational elements. In: Limbo R Wool C Carter BS, editors. Handbook of perinatal and neonatal palliative care: A guide for nurses, physicians, and other health professionals, chapter 4. New York, NY: Springer Company, LLC (2020). p. 50–65. 10.1891/9780826138422.0004

[19] Ludington-Hoe SM. Skin-to-skin contact: a comforting place with comfort food. MCN Am J Matern Child Nurs. (2015) 40:359–66. 10.1097/NMC.0000000000000178 26280947

[20] Karlsson V Heinemann A Sjörs G Nykvist KH Agren J. Early skin-to-skin care in extremely preterm infants: thermal balance and care environment. J Pediatr. (2012) 161:422–6. 10.1016/j.jpeds.2012.02.034 22497906

[21] Diekema DS. Botkin JR; Committee on Bioethics. Clinical report—forgoing medically provided nutrition and hydration in children. Pediatrics. (2009) 124:813–22. 10.1542/peds.2009-1299 19651596

[22] Parravicini E. Neonatal palliative care. Curr Opin Pediatr. (2017) 29:135–40. 10.1097/MOP.0000000000000464 28092282

[23] Parravicini E Daho M Foe G Steinwurtzel S Byrne M. Parental assessment of comfort in newborns affected by life-limiting conditions treated by a standardized neonatal comfort care program. J Perinatol. (2018) 38:142–7. 10.1038/jp.2017.160 29048412

[24] Llerena A Tran K Choudhary D Hausmann J Goldgof D Sun Y Neonatal pain assessment: do we have the right tools? Front Pediatr. (2023) 10:1022751. 10.3389/fped.2022.1022751 36819198 PMC9932268

[25] Carter BS Jones PM. Evidence-based comfort care for neonates towards the end of life. Semin Fetal Neonatal Med. (2013) 18:88–92. 10.1016/j.siny.2012.10.012 23182618

[26] Garten L Bührer C. Pain and distress management in palliative neonatal care. Semin Fetal Neonatal Med. (2019) 24(4):101008. 10.1016/j.siny.2019.04.008 31056417

[27] Cortezzo DE Meyer M. Neonatal end-of-life symptom management. Front Pediatr. (2020) 8:574121. 10.3389/fped.2020.574121 33042931 PMC7516797

[28] Haug S Farooqui S Wilson CG Hopper A Oei G Crater B. Survey on neonatal end-of-life comfort care guidelines across America. J Pain Symptom Manage. (2018) 55:979–84. 10.1016/j.jpainsymman.2017.10.023 29129740

[29] Janvier A Farlow B Barrington KJ. Parental hopes, interventions, and survival of neonates with trisomy 13 and trisomy 18. Am J Med Genet C Semin Med Genet. (2016) 172:279–87. 10.1002/ajmg.c.31526 27550159

[30] Carter BS. Pediatric palliative care in infants and neonates. Children (Basel. (2018) 5(2):e21. 10.3390/children5020021 PMC5835990 29414846

[31] De Lisle-Porter M Podruchny AM. The dying neonate: family-centered end-of-life care. Neonatal Netw. (2009) 28:75–83. 10.1891/0730-0832.28.2.75 19332405

[32] Committee on Fetus and Newborn, Section on Anesthesiology and Pain Medicine. Prevention and management of procedural pain in the neonate: an update. Pediatrics. (2016) 137:e20154271. 10.1542/peds.2015-4271 26810788

[33] Mangat AK Oei JL Chen K Quah-Smith I Schmölzer GM. A review of non-pharmacological treatments for pain management in newborn infants. Children (Basel). (2018) 5(10):130. 10.3390/children5100130 30241352 PMC6210323

[34] Woolf-King SE Anger A Arnold EA Weiss SJ Teitel D. Mental health among parents of children with critical congenital heart defects: a systematic review. J Am Heart Assoc. (2017) 6(2):e004862. 10.1161/JAHA.116.004862 28151402 PMC5523775

[35] Callahan K Steinwurtzel R Bruarie L Schechter S Parravicini P. Early palliative care reduces stress in parents of neonates with congenital heart disease: validation of the “baby, attachment, comfort interventions.”. J Perinat. (2019) 39:1640–7. 10.1038/s41372-019-0490-y 31488903

